# Face-Evoked Steady-State Visual Potentials: Effects of Presentation Rate and Face Inversion

**DOI:** 10.3389/fnhum.2012.00316

**Published:** 2012-11-27

**Authors:** L. Forest Gruss, Matthias J. Wieser, Stefan R. Schweinberger, Andreas Keil

**Affiliations:** ^1^Department of Psychology, Center for the Study of Emotion and Attention, University of FloridaGainesville, FL, USA; ^2^Department of Psychology, University of WürzburgWürzburg, Germany; ^3^DFG Research Unit Person Perception, Friedrich Schiller UniversityJena, Germany

**Keywords:** electroencephalography, face inversion effect, face processing, steady-state visually evoked potentials, N170, frequency-dependency

## Abstract

Face processing can be explored using electrophysiological methods. Research with event-related potentials has demonstrated the so-called face inversion effect, in which the N170 component is enhanced in amplitude and latency to inverted, compared to upright, faces. The present study explored the extent to which repetitive lower-level visual cortical engagement, reflected in flicker steady-state visual evoked potentials (ssVEPs), shows similar amplitude enhancement to face inversion. We also asked if inversion-related ssVEP modulation would be dependent on the stimulation rate at which upright and inverted faces were flickered. To this end, multiple tagging frequencies were used (5, 10, 15, and 20 Hz) across two studies (*n* = 21, *n* = 18). Results showed that amplitude enhancement of the ssVEP for inverted faces was found solely at higher stimulation frequencies (15 and 20 Hz). By contrast, lower frequency ssVEPs did not show this inversion effect. These findings suggest that stimulation frequency affects the sensitivity of ssVEPs to face inversion.

## Introduction

The human face is a ubiquitous social cue that engages specialized and highly efficient brain processes, involving different neural systems including the visual cortices (Haxby et al., 2000). An abundant research literature using functional imaging measures has identified different visual areas that are involved in face processing located around anatomically well-defined areas like the superior temporal sulcus (STS, e.g., Kanwisher et al., 1997; Gauthier and Logothetis, 2000), the middle fusiform gyrus (“Fusiform Face Area,” FFA, e.g., Puce et al., 1995; Kanwisher et al., 1997), and more posteriorly in the lateral part of the inferior occipital lobe (“Occipital Face Area,” OFA e.g., Gauthier and Logothetis, 2000). These areas are larger in size and generally show stronger and more consistent face preferential responses in the right than in the left hemisphere (e.g., Sergent et al., 1992; Kanwisher et al., 1997; Fox et al., 2009).

Electrophysiological studies have suggested that faces compared to non-face objects differentially engage visual sensory processing as early as at the stage of the posterior N1 component (∼150–200 ms latency) of the visual event-related potential (ERP). The face-sensitive response is usually referred to as N170, peaks between 140 and 200 ms after stimulus onset over lateral occipito-temporal cortex, and is typically more pronounced over the right hemisphere (Bentin et al., 1996; Eimer, 2000a; Halgren et al., 2000; Itier and Taylor, 2004). Source localization of the N170 or its magneto-encephalographic counterpart, the M170, point to ventral occipito-temporal structures including the lateral occipito-temporal sulcus area, the posterior fusiform gyrus, and the posterior STS, as well as inferior occipital sources as likely generators (Schweinberger et al., 2002; Itier and Taylor, 2004; Deffke et al., 2007).

A well-established finding in how face perception can modulate the visual processing of the stimulus is the so-called face inversion effect. In ERP studies, when viewing an upside-down face, the N170 has greater latency and negativity than for upright faces. A vast amount of literature has suggested this inversion effect to be face specific (Bentin et al., 1996; Eimer, 2000a,b; Rossion et al., 2000; Sagiv and Bentin, 2001; Itier et al., 2007), although Gauthier et al. (1999) interpreted this effect to be bound to expertise. Wiese et al. (2009) provided evidence for the specificity of this effect to human faces, in that both faces of same and different ethnicities resulted in the inversion effect of an enhanced N170. Inversion effects were absent however for faces of other species or houses. It is assumed that the enlarged N170 amplitudes in response to inverted compared to upright faces reflects the recruitment of additional resources due to the increased difficulty of processing inverted faces (e.g., George et al., 1996; Rossion et al., 1999) or the additional recruitment of eye-selective neurons (Itier et al., 2007; Itier and Batty, 2009) or object-selective neurons (Rossion et al., 1999, 2000) by inverted faces. On a cognitive level, the face inversion effect has been proposed to result from disruption of the processing of configural information (Yin, 1969; Valentine, 1988), making identification, and discrimination more difficult. Loss of configural information is conceptualized as being more important for face perception than for the perception of other visual objects (Carey and Diamond, 1977; Tanaka and Farah, 1993; Maurer et al., 2002).

In addition to time-locked ERPs, recent work in the cognitive and affective neurosciences has increasingly used the steady-state visual evoked potential (ssVEP) to study different aspects of face processing, including processing of emotional expression as well as face identification (e.g., McTeague et al., 2011; Rossion and Boremanse, 2011). The ssVEP is an oscillatory response to luminance- or contrast-modulated stimuli (Regan and Spekreijse, 1986), in which the frequency of the electrocortical response recorded from the scalp mirrors the driving frequency, often including higher harmonics. The oscillatory ssVEP is precisely defined in the frequency domain as well as the time-frequency domain. Thus, it can be reliably separated from noise and quantified as the evoked spectral power in a narrow frequency range, particularly at the frequency of the driving stimulus. Importantly, ssVEPs reflect multiple excitations of the visual system with the same stimulus over an extended epoch. The flicker-evoked ssVEP is predominantly generated in primary visual and to some extent in adjacent, higher order, cortices (Müller et al., 1997). Depending on stimulus presentation – or experimental design – the ssVEP can be driven in lower-tier visual cortices using high contrast luminance modulation with square-wave stimulation, or an alternate sinusoidal stimulation can be used to drive the ssVEP response in higher order cortices such as the fusiform cortex (Rossion and Boremanse, 2011).

A body of recent studies on emotion and attention has capitalized on ssVEP work in the area of selective attention, in which flicker-evoked ssVEPs, generated in lower-tiers of the visual system (Müller et al., 1997), are strongly modulated by experimental manipulations (Morgan et al., 1996; Hillyard et al., 1997; Andersen et al., 2009): both the amplitude and phase (latency) of the sensory electrocortical response as measured by ssVEPs have been shown to vary in response to both physical stimulus properties and subsequent re-entrant, top-down modulation of sensory activity via higher order processes (Silberstein et al., 1995; Keil et al., 2009). Enhancement of flicker ssVEP amplitudes has been observed as a function of visual spatial selective attention (Müller et al., 2003), fear conditioning (Moratti and Keil, 2005; Moratti et al., 2006), emotional stimulus content (Keil et al., 2003), as well as configural processing of complex visual information (Wang et al., 2007). Other than ssVEP studies in vision research, this body of work has often used complex stimuli and multiple complex objects – including faces – tagged at multiple stimulation frequencies, resulting in various challenges regarding experimental design, data reduction, and data analysis. One limitation of such cognitive ssVEP research is that little is known about the sensitivity of ssVEPs to basic manipulations of the complex stimulus material *per se*. This makes the interpretation of interactions with cognitive and emotional factors difficult.

Given the increased use of ssVEPs with face stimuli in studies of cognition and emotion, the present research aims to identify the extent to which the flicker ssVEP in a typical experimental design is sensitive to face inversion and how such sensitivity may vary as a function of presentation frequency. A series of studies from our laboratory (McTeague et al., 2011; Wieser and Keil, 2011; Wieser et al., 2011) has suggested that emotional expression modulates the ssVEP evoked by faces flickering at frequencies at and above 14 Hz, only in individuals with very high social anxiety, whereas participants drawn from the general population did not show expression-related effects. Consistent with work using flickering gratings, beamformer-based source projections estimated the ssVEP elicited by flickering faces to have an early visual (predominantly calcarine) origin (Wieser and Keil, 2011). This suggests that re-entrant feedback from higher cortices, well-established in animal and human work (Martinez et al., 1999; Roelfsema, 2006), may modulate lower-tier visual areas as a function of attention or motivational relevance of a stimulus. Here we examine the extent to which face inversion, likely processed in higher order cortices, also affects the processing reflected in flicker ssVEPs generated in lower-tier visual areas and what role, if any, driving frequency may play.

In an effort to expand our knowledge of the flicker ssVEPs sensitivity to cortical face processing, we set out to explore two main questions with this experiment: firstly, it was investigated to what extent the ssVEP amplitude elicited by faces is sensitive to face inversion and is affected in the same fashion as the N170 of the ERP (i.e., face inversion effect). This question has methodological implications in that it informs the use of ssVEP in face processing, but is also of conceptual interest, given the sustained character of the visual cortical engagement elicited by ssVEPs. Research examining indices of connectivity (Keil et al., 2009) and multimodal imaging (Di Russo et al., 2007) have suggested that the ssVEP signal is affected by both initial sensory processing and by re-entrant modulation originating in higher order occipito-temporal visual areas. In the present research, the disruption of the face configuration by inversion, which typically results in an N170 amplitude enhancement, may engage such top-down processes, hypothesized to facilitate correct encoding of the stimulus (Eimer, 2000a,b). Thus, we expected an amplification of the flicker ssVEP for inverted compared to upright faces due to re-entrant modulation from higher order visual cortices. We did not expect differences in emotional expression or attention effects to significantly affect the ssVEP amplitude in the current study. As an added control, we measured ERP components such as the P1 and N170 evoked by the onset of the face stream. Although these measures are limited due to the small number of trials used and the oscillatory nature of the stimulus stream, we were interested in replicating the face inversion effect with this non-traditional N170. Furthermore, in a set of *post hoc* analyses, we examined the extent to which N170 and ssVEP inversion effects co-vary across participants. This opens an avenue to exploring the degree to which ssVEP modulation is related to N170 effects.

As far as predictions of the role of driving frequency plays on face processing, in particular face inversion using flicker-evoked ssVEP, it is difficult to predict an outcome as there is no singular model of ssVEP generation. Many aspects of the ssVEP can be modeled by linear superposition of single transient ERPs (Capilla et al., 2011). It is conceivable that such superposition may lead to destructive interference at certain frequencies, where components sensitive to face inversion are canceled out by interacting with previous or subsequent components overlapping in time (Heinrich, 2010). By the same token, other frequencies may be particularly beneficial in amplifying face inversion effects, where for instance the N170 of a previous flicker may superimpose on the subsequent N170 (constructive interference). Empirically, driving frequency has been shown to play a role in some (Ding et al., 2006), but not all conceptual domains (Keitel et al., 2010) studied by means of ssVEP. To determine the extent to which face-evoked ssVEPs display differential inversion sensitivity for different stimulation rates, we used four different driving frequencies, presenting faces at 5, 10, 15, and 20 Hz. The interplay of linear versus non-linear effects is difficult to delineate, therefore we do not make any strong speculations in such an exploratory study on what effects we predict using these frequencies. It is of particular interest though exploring frequencies in the beta range, as these are commonly used in cognitive ssVEP studies.

## Materials and Methods

### Participants

Twenty-three undergraduate students from the University of Florida participated for psychology course credit in Experiment 1. Two data sets were rejected due to excessive noise, resulting in 21 participants (18 female; 13 Caucasian; 20 right handed) ranging in age from 18 to 23 years (*M* = 19.2; SD = 1.3). Twenty-one undergraduate students from the University of Florida participated for psychology course credit in Experiment 2. Three data sets were rejected due to excessive noise, resulting in 18 participants (12 female; 14 Caucasian; 17 right handed) ranging in age from 18 to 21 (*M* = 18.5; SD = 0.9). All participants had normal or corrected vision and reported no personal or family history of seizures.

### Stimuli

Ninety-six pictures from the Karolinska Directed Emotional Faces (KDEF) database (Lundqvist et al., 1998) were selected, consisting of 24 actors (12 female, 12 male) with frontal gaze, displaying four emotional expressions (neutral, happy, angry, and fearful). To enable comparisons with previous and ongoing work employing emotional face stimuli, the present research used four different emotional expressions, although no differences between expressions were expected in our healthy sample. Pictures of the KDEF are standardized with respect to eye position, which is at the vertical midline of the picture. In addition, all 96 images were rotated by 180°. This resulted in having a total of 192 images, half of which were upright faces and the other half inverted faces. All images were gray scaled and adjusted for average luminance, computed using the Matlab image processing toolbox. Face pictures were sized at 281 × 381 pixels, and spanned a visual angle of 3.9° horizontally and 5.4° vertically.

### Design and procedure

Upon arrival at the laboratory, participants provided informed consent, were seated in a sound-attenuated, electrically shielded chamber, and told they would be viewing a series of flickering upright and inverted faces of different emotional expressions. The sensor net was applied and participants were instructed to maintain constant and active gaze on the stimuli and reduce eye blinks and head movements to a minimum. Stimuli were presented on a 21″ CRT monitor with a vertical retrace rate of 60 Hz, 1.5 m away from the seated participant in the darkened chamber. Each stimulus was displayed in a flickering (square-wave) fashion in the center of the screen with a black background for a trial length of 4000 ms (Experiment 1) or 3000 ms (Experiment 2), using Psychtoolbox running on Matlab (Brainard, 1997). Two flicker rates were used for each experiment, a slow and a fast rate. The need for multiple frequencies – often a need in studies in which frequency tagging with multiple rates is desired – made it impractical to fully adhere to the helpful guidelines for ssVEPs in vision research (Bach and Meigen, 1999), although these provided guidance where possible. Stimulation epochs were designed to contain integer number of cycles and only frequencies allowing for the exact same epoch duration (thus differing in the number of cycles) were compared within the same group of participants: experiment 1 had 5 Hz (flicker cycle = 200 ms; picture on for 100 ms, picture off for 100 ms; 20 total cycles) and 15 Hz (flicker cycle = 66.67 ms; picture on for 33.34 ms, picture off for 33.34 ms; 60 total cycles). Experiment 2 had 10 Hz (flicker cycle = 100 ms; picture on for 50 ms, picture off for 50 ms; 30 total cycles) and 20 Hz (flicker cycle = 50 ms; picture on for 16.67 ms, picture off for 33.34 ms; 60 total cycles). This procedure resulted in four different conditions for stimuli presentation: upright and inverted faces at slow and fast frequencies. Only one face was presented in a given trial, in a flickering fashion as described above. Between trials a fixation cross was present for the participant to maintain gaze. With 192 trials, a variable inter trial interval of 2–4 s, as well as a short break in the middle, the EEG recording lasted approximately 24 min (Experiment 1) or 21 min (Experiment 2) after which participants were debriefed. This allowed for 24 trials per condition, per frequency, to be collected in a short time frame of 20–25 min, rather than a 45 min recording to collect all four frequencies per individual. All procedures were approved by the institutional review board of the University of Florida.

### EEG recording and data collection/processing

Electroencephalogram (EEG) was continuously recorded from 257 electrodes using an Electrical Geodesic (EGI) sensor net, with Cz as the recording reference. EEG was digitized at 250 Hz, band-pass filtered online between 0.1 and 50 Hz and impedances were kept below 60 kΩ. Offline, recorded data were low-pass filtered at a frequency of 40 Hz. For Experiment 1, 5400 ms epochs were extracted (600 ms pre- and 4800 ms post-stimulus onset), 4200 ms epochs for Experiment 2 (600 ms pre- and 3600 ms post-stimulus onset). Artifact rejection was subsequently implemented through EMEGS software (Peyk et al., 2011). This procedure detects artifacts in individual recording channels using the recording reference (Cz), based on the distribution of the mean, standard deviation, and gradient of the voltage amplitude. Data are then converted to average reference, and global artifacts (e.g., trials with blinks and/or movement artifacts) are eliminated. In addition, we monitored deviation from fixation (during the ITI) using vEOG and hEOG and trials with indication of eye movements were discarded. Individual sensors contaminated with artifacts were interpolated using a statistically weighted, spherical spline interpolation from the full channel set. Trials of the same condition were averaged together to form condition-specific time domain representations of evoked activity. After artifact correction, an average of 70% (Experiment 1) and 68% (Experiment 2) of the total trials were retained for further analyses. Trigger pulses synchronized to the screen retrace were sent through Psychtoolbox to the EEG amplifier and co-registered with the EEG. Timing accuracy and reliability was tested with a photo-diode. Additional synchronization of the screen, the trigger, and the amplifier digitization was not possible with the equipment used.

### SSVEP and ERP analyses

**Figure 1:**
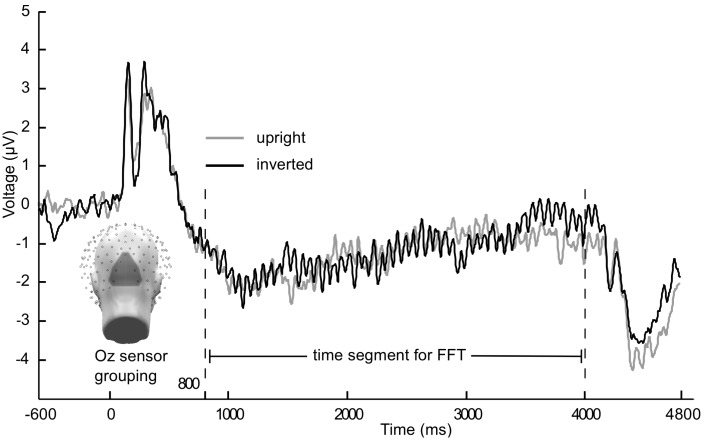
**Grand mean (*n* = 21) time course of the steady-state visually evoked potential**. Averaged across Oz sensor grouping over occipital pole for 15 Hz flicker condition from Experiment 1. Gray lines are upright faces, black lines are inverted faces.

**Figure 2:**
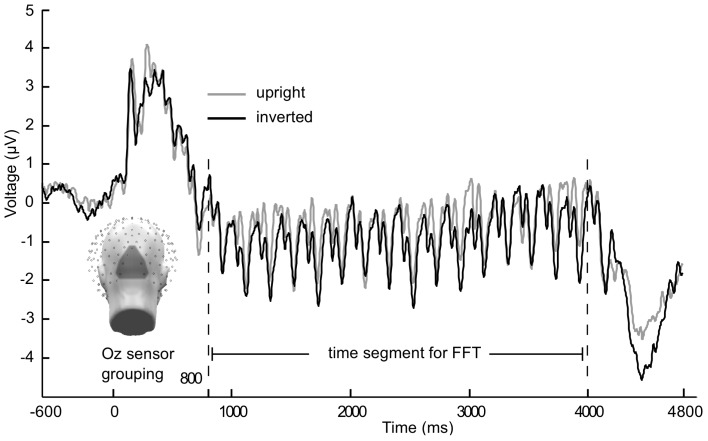
**Grand mean (*n* = 21) time course of the steady-state visually evoked potential**. Averaged across Oz sensor grouping over occipital pole for 5 Hz flicker condition from Experiment 1. Gray lines are upright faces, black lines are inverted faces. Caution is warranted with observing multiple frequencies in the time domain averaged data, as grand mean may include differing phases (across participants) and conversion into the frequency domain along with statistical analyses is necessary before making inferences about multiple frequencies contained in the signal.

Each of the four stimulus frequencies resulted in their ssVEP having the same fundamental frequency as the driving frequency. A Discrete Fourier Transformation (DFT) analysis was conducted for each condition and subject on trial-averaged data, showing pronounced peaks in the frequency spectrum at the flicker frequency. This was done using the fft function implemented in Matlab, applied to ssVEP segments as described in the following paragraph. As trial lengths differed from Experiment 1 to Experiment 2, two different segment lengths were chosen: 800–4000 ms after stimulus onset for Experiment 1, 800–3000 ms for Experiment 2. The first 800 points were excluded to avoid contamination with the ERP to the stream onset, as done in previous work. To allow for better comparison of the spectra across experiments, data from Experiment 2 was zero padded to have the same frequency resolution (0.3125 Hz) as Experiment 1. This also allowed us to average spectra for illustration and topographical analysis across multiple frequencies. A disadvantage of this approach is the lower power resulting from adding zeros in Experiment 2. This shortcoming was addressed by comparing the results with an analysis on non-padded data^1^. Main effects of experiment were not of interest in the present mixed-model design aiming to explore interactive effects between frequency and face inversion. To obtain an accurate spectral representation, refresh rate of the monitor, sampling rate, and cycle counts of each frequency were taken into consideration when selecting the appropriate time segment for the Fourier transform. This is in line with previous work from our laboratory (e.g., Keil et al., 2008) as well as with the recommendations by Bach and Meigen (1999). Given the sampling rate of 250 Hz, one sample point was recorded every 4 ms, therefore resulting in a 3200 ms epoch (Exp 1) that contained 800 sample points (from sample point 351 to 1150). In Experiment 2, as the epoch was 2200 ms long, (550 sample points, from 351 to 900) 1000 ms were added as zeros, i.e., 250 sample points. Integer number of complete cycles per frequency contained within the epochs extracted for each experiment are as follows: 16 cycles for 5 Hz, 22 cycles for 10 Hz, 48 cycles for 15 Hz, and 44 cycles for 20 Hz. Fourier coefficients were obtained through the fft function in Matlab, and then normalized by the number of points. The amplitude spectrum was then extracted as the absolute value of the Fourier coefficients. Frequency spectra therefore resulted in a length of 400 frequency bins, with single bins being selected per frequency condition: bin 17 (5 Hz), bin 33 (10 Hz), bin 49 (15 Hz), and bin 65 (20 Hz). Data were then averaged across a cluster of ten sensors, including Oz and POz, and the resulting mean was used for further ssVEP analysis (see Figure 1). In addition, second, and where possible third, harmonics were analyzed in the same manner for frequency conditions 5, 10, and 15 Hz. The second harmonic of the 20 Hz condition was not analyzed due to our filter occurring at 40 Hz. For all analyses of the ssVEP, raw amplitudes were used. P1 analysis used a similar, slightly more superior, sensor grouping as for the ssVEP. For N170 analysis, two symmetrical sensor groupings were used for left and right hemispheric activity at lateral occipito-temporal locations (including PO7 and P9 and PO8 and P10, respectively). To obtain P1 and N170 amplitudes to the onset of the stimulus train, all latencies were manually inspected and time windows were selected, 24 ms for the P1 and 32 ms for the N170. Peak values within these time windows were then extracted after a 100 ms baseline correction, resulting in unique amplitude and latency values per individual. Amplitudes were then averaged across subjects in the appropriate sensor groupings (see Figures 1 and 2 for grand mean averaged onset ERPs in Experiment 1).

### Statistical analyses

In a first step, ANOVAs were conducted on ssVEP and ERP P1/N170 amplitude means, derived from the sensor groupings previously described. Within-subject factors for the ssVEP ANOVA were orientation (upright, inverted faces), presentation rate (slow, fast), emotion (happy, neutral, angry, fearful). A between-subject factor of experiment (1, 2) was included as well. For the P1 and N170 ANOVAs, the factors orientation, presentation rate, and experiment were used, with the addition of a within-subject factor of hemisphere (left, right) for the N170. This factor was included because previous research has shown hemispheric differences, with the right hemisphere in particular showing a greater N170 response to faces than the left hemisphere (Bentin et al., 1996; Eimer, 2000a; Halgren et al., 2000; Itier and Taylor, 2004). In a second step, difference amplitudes (between orientations) of ssVEP and ERP components were related using Pearson product-moment correlation coefficients, to test the extent to which any ssVEP increases for inverted faces are a linear function of relative N170 enhancements. These exploratory correlation analyses were done comparing the same electrode sites, as well as correlating across locations (occipito-temporal versus Oz grouping), to account for the fact that N170 and ssVEP were measured at these two different sites.

## Results

In Figures 1 and 2 the averaged grand mean posterior ssVEPs are depicted for both the 15 and 5 Hz condition of Experiment 1, respectively.

**Figure 3:**
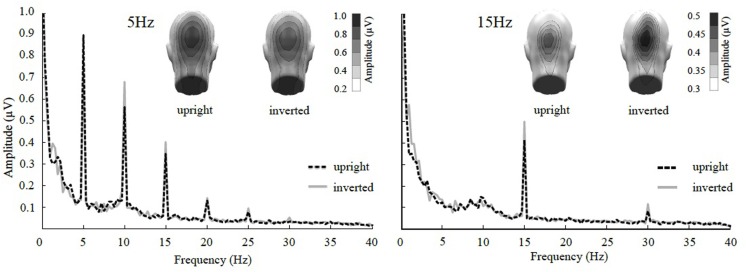
**Frequency spectrum at Oz together with topographical maps of evoked power at the stimulus frequencies of Experiment 1**. SsVEP amplitude increase to inversion is significant for the 15 Hz and its 30 Hz harmonic, but not for the 5 Hz condition nor any of its harmonics.

**Figure 4:**
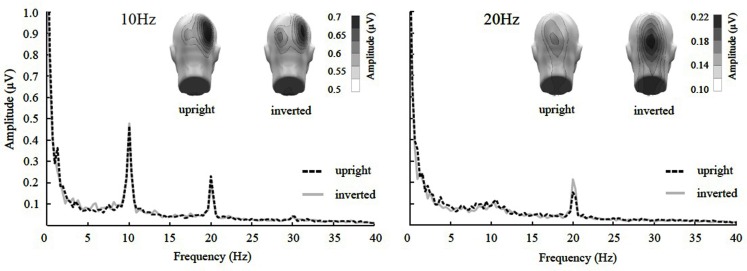
**Frequency spectrum at Oz together with topographical maps of evoked power at the stimulus frequencies of Experiment 2**. SsVEP amplitude increase to inversion is significant for the 20 Hz, but not for the 10 Hz condition nor its 20 Hz harmonic.

**Figure 5:**
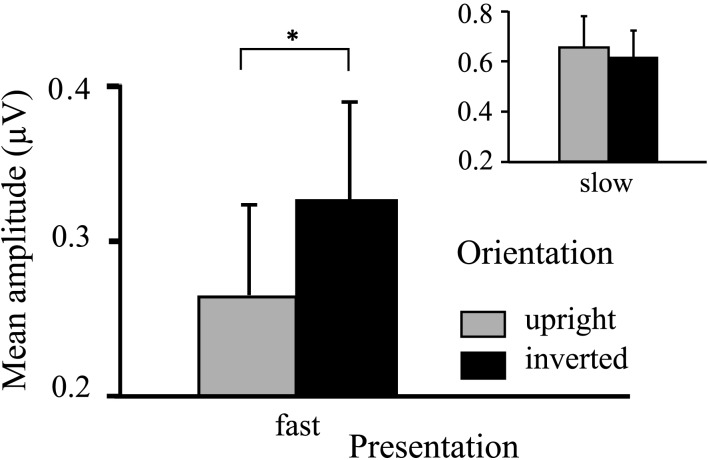
**Graph displaying interaction effect of presentation rate**. Faster frequencies in both experiments (i.e., 15, 20 Hz) show overall increased ssVEP amplitude for inverted faces, whereas slower frequencies in both experiments (i.e., 5, 10 Hz) do not show this. Error bars indicate SEMs for ssVEP amplitudes.

The ANOVA revealed a main effect of presentation rate, in that slower frequencies in both experiments showed greater amplitudes than the faster frequencies [*F*(1, 37) = 28.60, *p* < 0.001]. In addition, an interaction of presentation rate × orientation showed that only in the faster frequencies the inverted faces showed greater amplitude enhancement [*F*(1, 37) = 6.71, *p* < 0.05; see Figures 3–5]. *Post hoc* ANOVAs showed this to be true in the 15H z [*F*(1, 20) = 9.82, *p* < 0.01] and the 20 Hz condition [*F*(1, 17) = 12.24, *p* < 0.01], but not in the 5 and 10 Hz condition. In addition, the frequency spectra are displayed in Figures 3 and 4 for each driving frequency at Oz, illustrating the high SNR as well as resulting harmonics. SNR values are as followed: 7.8 (5 Hz), 4.8 (10 Hz), 8.2 (15 Hz), and 3.2 (20 Hz). Separate *t*-tests conducted on the harmonics of 5 and 10 Hz ssVEPs revealed no significant differences between upright and inverted faces. The second harmonic of the 15 Hz condition revealed a significant inversion effect [*t*(20) = −2.48, *p* < 0.05], identical to the fundamental driving frequency. The 20 Hz condition was not analyzed for harmonics due to the low-pass filter set to 40 Hz.

Mean ssVEP amplitudes also differed between both experiments, in that Experiment 2 (using higher driving frequencies) had overall smaller amplitudes [*F*(1, 37) = 6.56, *p* < 0.05; see Table 1 for mean ssVEP amplitudes across all frequencies]. Across dependent variables, no main effect or interaction was found involving emotional expression, and therefore all expression conditions were collapsed for all subsequent analyses. The P1 amplitude did not vary with the experimental manipulations.

**Table 1:** **Mean ssVEP amplitudes (μV) across participants with SEM, averaged across 10 occipital sensors including Oz and POz, for both upright and inverted faces for all frequency conditions**.

	5 Hz	10 Hz	15 Hz	20 Hz
Upright faces	0.82 (0.14)	0.47 (0.08)	0.38 (0.07)	0.13 (0.02)
Inverted faces	0.73 (0.12)	0.49 (0.08)	0.44 (0.07)	0.19 (0.03)

**Figure 6:**
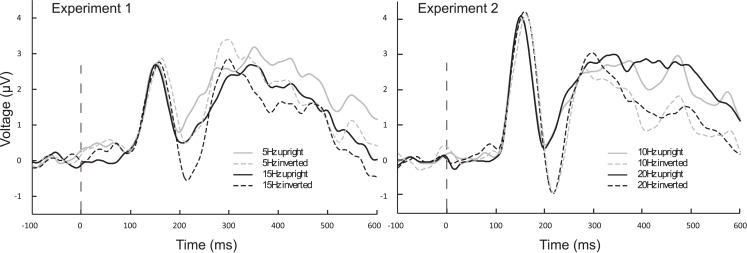
**Averaged ERPs from left and right hemispheric, occipito-temporal sensor groupings, to onset of stimulus train**. ERPs illustrate that inverted (dashed lines) conditions differ from one another only in Experiment 1, where the 5 Hz N170 is diminished in compared to all other inverted conditions. Black lines are faster frequencies, gray lines are slower frequencies, and solid lines are the upright conditions.

The ANOVA for the N170 revealed a main effect of presentation rate, in that the N170 was less negative in the slower frequencies [*F*(1, 37) = 5.99, *p* < 0.05], as well as a main effect of orientation, with an N170 enhancement for inverted faces [*F*(1, 37) = 21.35, *p* < 0.001]. Furthermore, a three-way interaction of orientation × hemisphere × experiment was found [*F*(1, 37) = 6.15, *p* < 0.05]. *Post hoc* ANOVAs further exploring this three-way interaction indicated that with inversion, N170 was enhanced over the right hemisphere for Experiment 2, but not for Experiment 1 [*F*(1, 37) = 4.50, *p* < 0.05], and that this effect was specific to the fast presentation rate [*F*(1, 37) = 4.13, *p* < 0.05] (more represented in Experiment 2). A further *post hoc* ANOVA showed that the N170 of the slow versus fast presentation rate differed significantly from one another in the inverted conditions only in Experiment 1 [*F*(1, 20) = 5.56, *p* < 0.05], but not in Experiment 2. Figure 6 illustrates this, in that the N170 to inverted faces in the 5 Hz condition is less pronounced in comparison to the other frequency conditions, irrespective of hemisphere. See Table 2 for a breakdown of mean N170 amplitudes across all frequencies from left and right hemispheric sensor groups.

**Table 2:** **Mean N170 amplitudes (μV) across participants with SEM, averaged across a left (11 sensors including PO7 and P9) and a right (10 sensors including PO8 and P10) sensor grouping, for both upright and inverted faces for all frequency conditions**.

		5 Hz	10 Hz	15 Hz	20 Hz
Upright	Left	0.70 (0.32)	0.89 (0.32)	1.43 (0.62)	0.72 (0.32)
faces	Right	0.68 (0.32)	0.93 (0.36)	1.44 (0.58)	1.16 (0.41)
Inverted	Left	−0.43 (0.42)	−0.05 (0.30)	0.83 (0.34)	−0.17 (0.34)
faces	Right	−0.31 (0.45)	−0.72 (0.45)	0.48 (0.43)	−0.69 (0.40)

In addition, a two-way interaction of presentation rate × experiment revealed less negative N170 values for the slow presentation rates in Experiment 1, whereas data from Experiment 2 did not exhibit a difference between presentation rates [*F*(1, 37) = 6.80, *p* < 0.05]. Mean N170 amplitudes also differed significantly between experiments, Experiment 2 having greater N170 negativity overall [*F*(1, 37) = 6.32, *p* < 0.05].

In a *post hoc* analysis aiming to explore the relationship between N170 and ssVEP modulation across subjects, correlation analyses on inversion-related difference amplitudes for the ssVEP and ERP were conducted. Since inversion effects result from different directions for the two measures (more negative values indicating a greater inversion effect for the N170, whereas more positive values indicating a greater inversion effect for the ssVEP amplitudes), difference amplitudes were obtained in two separate ways: N170 amplitude of upright minus inverted faces; ssVEP amplitude of inverted minus upright faces. As a result, positive values indicated that the measures were greater for inverted faces. Two separate analyses were conducted, one correlating the N170 values from two occipital temporal sensor clusters (as described in the Materials and Methods) to the ssVEP at the previously stated occipital pole cluster, and another with same site correlation at the occipital pole cluster for N170 and ssVEP. Only one of the Pearson product-moment correlation coefficients proved to be statistically significant (*p* < 0.05) with a positive correlation between the two values (0.547): right hemispheric N170 to 20 Hz ssVEP amplitude. This positive correlation indicated that participants showing a 20 Hz face inversion effect also showed a pronounced right hemispheric N170 inversion effect (left N170 trending with a coefficient of 0.460). Such a positive correlation was neither found for any other frequency condition, nor for the P1 values.

## Discussion

The current study examined the extent to which the face-evoked ssVEP is sensitive to face inversion, and how such sensitivity may depend on the stimulation frequency. Two separate experiments were conducted with a total of four different presentation rates (5, 10, 15, and 20 Hz). SsVEP amplitude was found to be enhanced for the inverted, compared to upright faces, solely at faster presentation rates (i.e., 15 and 20 Hz) an effect that was consistent across two groups of participants. No such increase was found when faces were flickered at rates of 5 and 10 Hz. The results therefore support the notion of a frequency-dependency of the face inversion effect in the ssVEP.

As a manipulation check, and to examine the relationship among different measures of face inversion, we extracted the ERP components elicited by the face stream onset. Importantly, the typical N170 face inversion effect was replicated in the current study, reliably showing enhanced negativity to inverted versus upright faces. A right hemispheric preponderance of the N170 to inverted faces was found in conditions with faster frequencies. This is in line with the notion that the right hemisphere tends to be more affected by configurational disruption of the stimulus (Bradshaw and Sherlock, 1982; Rhodes, 1993). Current research by Mohamed et al. (2011) suggests that face inversion impairs the interactive encoding of hierarchical cues (e.g., body, head/face, and eyes) across multiple cortex areas (e.g., fusiform and extrastriate body areas, fusiform and occipito-temporal face areas).

The combined findings that both ssVEP and N170 show a strong face inversion effect, specifically at higher presentation rates, raises the question as to what mechanisms are driving this frequency-dependent inversion effect in ssVEP and ERP modulation during face processing. One popular hypothesis of how ssVEPs are generated assumes that they are linear superpositions of individual transient evoked potentials (Capilla et al., 2011). It should be noted however that starting with early work (see Regan and Spekreijse, 1986), strong evidence has been presented for non-linear effects contributing to the ssVEP (e.g., Liu et al., 2010). Assuming a linear model for the steady-state, we would expect face inversion effects of the ssVEP to be more pronounced in the slower conditions. In particular, in the 5 Hz condition each cycle allows a fully developed P1/N170 complex to emerge while the P3 of the immediately proceeding cycle is suppressed. Multiple P1/N170 complexes adding in such a linear fashion would then result in increased amplitude of the ssVEP at low enough frequencies. Yet, the inversion effect was only found for faster frequencies of the ssVEP, not supportive of a linear superposition model. The pronounced inversion effect of the N170 in conditions with higher frequency ssVEPs may be attributed to the fact that by the time the N170 has developed, multiple inverted faces have already been presented in the stimulus train. This may be suggestive of a non-linear facilitation effect contributing to the inversion effect of the N170 in conditions with higher frequency ssVEPs.

Heinrich (2010) purported that depending on repetition rate of the flickering stimulus, some components can be expected to be amplified and some to be eliminated by destructive interference. These processes would lead to differential sensitivity of the ssVEP to experimental manipulation as a function of what aspect of the transient evoked potential is retained, amplified, or eliminated. This uncertainty makes it difficult to determine the extent to which the current ssVEP results are due to superposition, linear, or non-linear in nature, of the N170. Furthermore, *post hoc* correlation analyses conducted to explore the covariation of N170 and ssVEP inversion effects found that although the N170 enhancement by inverted faces was linearly related to ssVEP amplitude enhancement in the 20 Hz condition, such a relationship was not found for any other frequency condition. Additionally, ongoing work in our laboratory, which will be described elsewhere, used formal computational modeling of the ssVEP by linear superposition of transient ERPs (Capilla et al., 2011). The results of this are not indicative of a linear relationship between the N170 and the ssVEP. Taken together, a linear superposition model is insufficient in explaining the results of the current study. Rigorous testing of a wide range of frequencies would be necessary to examine possible constructive and destructive interferences that may occur in ssVEPs in such a paradigm. Furthermore, it is conceivable that slower waveforms, such as the 5 Hz ssVEP, are more sensitive to destructive interference than faster frequencies. In a study by Rossion and Boremanse (2011), a 3.5 Hz ssVEP was used to examine differences in cortical responses to identical and different faces. Here inversion of the faces was utilized to show that due to configurational effects in facial identification, inverting the face abolishes any effect seen in the upright conditions. In addition, the absence of an inversion effect in the 10 Hz condition of the current study could be explained by the effects being masked when stimulating in the alpha range (i.e., 8–13 Hz), which is characterized by high ssVEP resonance (Herrmann, 2001), and engagement of a wider range of brain areas (Keil et al., 2006, 2008). Such wider distribution is also reflected in the present topography of the 10 Hz ssVEP, which shows different lateralization and broader distribution, possibly reflecting alpha resonance (Herrmann, 2001).

As an alternative to the linear superposition model, Regan (1989) suggested that the ssVEP may be regarded as a linear plus a non-linear process of superposition. In his theoretical analysis of the ssVEP, the interference between subsequent waves may result in the emergence and disappearance of experimental effects at different frequencies. In line with a non-linear explanation, one may speculate that the low-frequency ssVEP signal has time to travel out of the lower-tier visual cortex to higher order cortices within each cycle (Müller et al., 1997). With this dispersion, more crosstalk between cortical areas may be possible, potentially masking observable effects resulting from rapid re-entrant fusiform-calcarine interactions (Keil et al., 2009). By contrast, local interactions within the visual cortex might be highlighted at higher driving frequencies, in which each new afferent activation occurs before the signal has left the peri-calcarine area (Regan and Spekreijse, 1986).

The exploratory nature of this study opens a wide avenue of further inquiry to expand on our present conclusions. As expected based on earlier work (McTeague et al., 2011), varying the emotional expression did not result in reliable ssVEP amplitude differences for either upright or inverted faces in our non-selected student sample. Comparing a socially high anxious to a socially low anxious group might reveal expression-related differences, particularly in terms of how face inversion affects these two groups. In addition, future research can examine the frequency-dependency of face inversion effects in more detail: is there a preferred frequency where this effect is ideally expressed? The behavior of the face inversion effect along the frequency spectrum using ssVEP would not only inform further as to cortical sensitivity to face inversion as a whole, but may also shed light on ssVEP generation.

**Figure 7:**
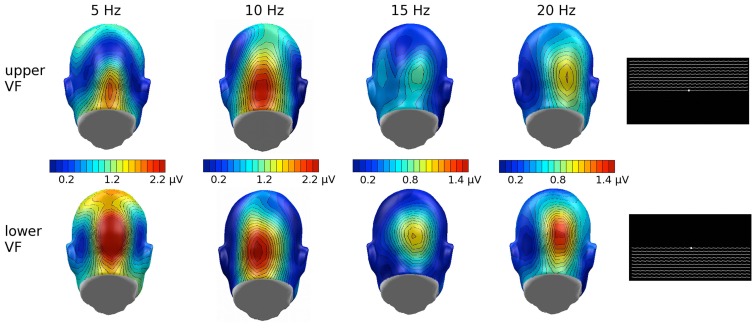
**Grand mean (*n* = 9) scalp topographies of the ssVEP response to the individual driving frequencies, in the upper and lower visual field (VF)**. Overall lower VF showed enhanced ssVEP amplitude and, relevant to the current study, no frequency-dependency found in this additional study.

With complex stimuli, we cannot rule out that any observed effect of inversion reflects physical differences between the upper and lower half of the face falling in different regions of the visual field. The Karolinska faces, when inverted, may result in higher luminance contrast between the black background and the gray background, in the lower visual field. To the extent that this interacts with stimulation frequency however, it seems an unlikely explanation, as effects of brightness and contrast on the flicker ssVEP tend to be relatively frequency independent, although they may vary with spatial frequency (Strasburger et al., 1988). To examine the effects of contrast changes in the upper versus lower visual field, with our experimental setup, we compared the impact of flickering upper visual field and lower visual field contrast stimuli (see Figure 7) in a sample of seven male student observers from the University of Florida, using the same timing and stimulus duration as reported in this study with the face stimuli, as well as the same instructions (passively observe) and analytic methods. In this *post hoc* analysis, the ssVEP in response to lower visual field luminance contrast tended to be more pronounced than upper visual field stimulation, and this effect did not change across driving frequencies. Wilcoxon signed-rank tests suggested that this difference was reliable for all driving frequencies, except for 10 Hz, which showed a trend in the same direction (*p* < 0.09). This is a different pattern than obtained for the face stimuli in the present study, where an interaction of driving frequency and inversion was observed. More systematic studies however are needed to address this important point. In the same vein, attention is known to affect the flicker ssVEP, but there are no previous reports that attention effects change in direction in the same way as reported here, as the driving frequency is altered (Keitel et al., 2010).

In summary, the frequency-dependency of the ssVEP seems to support that lower-tier visual cortical responses are affected by configural face processing. Caution is warranted because of the systematic physical differences of lower and upper halves of the faces, which reverse in their retinotopic position when faces are inverted. The present study is however a first step to establishing that the flicker ssVEP may show sensitivity to face configuration, and that such a sensitivity depends on the presentation rate.

## Conflict of Interest Statement

The authors declare that the research was conducted in the absence of any commercial or financial relationships that could be construed as a potential conflict of interest.

## Footnotes

^1^ssVEP analysis was also carried out on non-zero padded data for Experiment 2, including ANOVA analysis, and there were no differences in the outcome of the results, with all interaction effects –targeted in this study – being of comparable size in the analyses with padded and non-padded data.
